# Camera trap arrays improve detection probability of wildlife: Investigating study design considerations using an empirical dataset

**DOI:** 10.1371/journal.pone.0175684

**Published:** 2017-04-19

**Authors:** Kelly M. O’Connor, Lucas R. Nathan, Marjorie R. Liberati, Morgan W. Tingley, Jason C. Vokoun, Tracy A. G. Rittenhouse

**Affiliations:** 1 Wildlife and Fisheries Conservation Center, Department of Natural Resources and the Environment, University of Connecticut, Connecticut, United States of America; 2 Ecology & Evolutionary Biology, University of Connecticut, Storrs, Connecticut, United States of America; Cornell University, UNITED STATES

## Abstract

Camera trapping is a standard tool in ecological research and wildlife conservation. Study designs, particularly for small-bodied or cryptic wildlife species often attempt to boost low detection probabilities by using non-random camera placement or baited cameras, which may bias data, or incorrectly estimate detection and occupancy. We investigated the ability of non-baited, multi-camera arrays to increase detection probabilities of wildlife. Study design components were evaluated for their influence on wildlife detectability by iteratively parsing an empirical dataset (1) by different sizes of camera arrays deployed (1–10 cameras), and (2) by total season length (1–365 days). Four species from our dataset that represented a range of body sizes and differing degrees of presumed detectability based on life history traits were investigated: white-tailed deer (*Odocoileus virginianus*), bobcat (*Lynx rufus*), raccoon (*Procyon lotor*), and Virginia opossum (*Didelphis virginiana*). For all species, increasing from a single camera to a multi-camera array significantly improved detection probability across the range of season lengths and number of study sites evaluated. The use of a two camera array increased survey detection an average of 80% (range 40–128%) from the detection probability of a single camera across the four species. Species that were detected infrequently benefited most from a multiple-camera array, where the addition of up to eight cameras produced significant increases in detectability. However, for species detected at high frequencies, single cameras produced a season-long (i.e, the length of time over which cameras are deployed and actively monitored) detectability greater than 0.75. These results highlight the need for researchers to be critical about camera trap study designs based on their intended target species, as detectability for each focal species responded differently to array size and season length. We suggest that researchers *a priori* identify target species for which inference will be made, and then design camera trapping studies around the most difficult to detect of those species.

## Introduction

Camera trapping (CT) has become a popular technique employed in the field of wildlife ecology that offers researchers an opportunity to survey wildlife populations in an economic and non-invasive manner over longer periods than traditional survey methods. As CT technology has become more affordable and widely available, the range of applications for CT data has dramatically expanded [1]. Qualitative CT studies offer a novel and unprecedented opportunity to monitor rare and cryptic species [2]. Large-scale monitoring efforts using CT data have become particularly prevalent in recent literature, with the majority of CT research focused on questions of species occupancy (i.e., detection/non-detection information) or the relative abundance of many species simultaneously [3, 4]. Remote cameras are a staple tool in both ecological research and wildlife management.

The rapid expansion of CT technology in wildlife research has come with some noted pitfalls and shortcomings. Inconsistencies in CT terminology paired with inadequate reporting of study design considerations leads to potential confusion and an inability to compare findings across CT studies [5]. For example, camera make, model, sensitivity, and positioning often go unreported, despite these settings having meaningful impacts on conclusions that can be drawn from collected data [6]. Disregarding how these variables influence data will limit the ability for CT studies to accurately and consistently address ecological questions of abundance, density, occupancy and multi-species interactions or community dynamics. The relative ease of CT implementation compared to traditional survey methods (e.g., point counts, line transects) does not excuse lack of thoughtful consideration for study design. Researchers designing CT studies must decide how to appropriately allocate resources to maximize both detection probability and a return on investment. Deciding how many cameras to deploy and the length of time cameras should be deployed (i.e., season length) are undoubtedly part of the study planning and design, however the justification behind these decisions are rarely reported. It is important that researchers *a priori* define their sites, the time period over which occurrence is assumed to be closed to change (i.e., closure assumption) [7, 8], and the criteria that constitutes detection of a species [9]. Further analytical designs must be considered for applications and analyses that rely on repeated surveys, such as occupancy models [10]. The benefit (and difficulty) of CT data is that cameras are able to monitor continuously within a user-defined study period; however continuous data can cause analytical complications that should be addressed in the study design. Ultimately, the rigorous design is balanced against decisions made about costs of equipment, labor needed to deploy and process images, the need to meet model assumptions of closure that are grounded in the ecology of target species, as well as an assumed desire to gain maximum spatial coverage of an area while also ensuring reasonable detectability of one or many species [9]. These study design decisions determine the type and strength of inference that can be derived from collected data and could potentially lead to incorrect conclusions if not thoroughly considered.

Detectability, the ability to detect a species based on its presence within a surveyed area, is of critical importance in any effort to monitor wildlife populations. Measures of occupancy, abundance, and density are all influenced by species’ detectability, yet detectability is often unaddressed or poorly handled in CT literature [6]. This is despite well-established analytical methods, such as occupancy modeling [8], which can address imperfect detection. Naive estimates (i.e., those that do not account for detectability) of occupancy parameters can result in severely biased results and ecologically incorrect conclusions [10, 11].

Equally important is how the choice of study design may impact estimates of detectability. The detectability of species is a key feature frequently used in determining the optimal allocation of sites versus repeat visits in occupancy studies [12], but study design choices are also at least partly responsible for the detectability of species. These study design considerations are particularly relevant to CT studies which often use non-random camera placements to increase detectability. Other efforts to increase detection probabilities include study designs with increased length of survey seasons, increased number of camera days (e.g., [13], but see comment by [14]), or the use of baits and lures. Here, we use multi-camera “arrays” consisting of clustered, non-independent groups of randomly placed cameras within a site (i.e. properties of interest separated by ≥10 km), and thus present a study design choice distinct from increasing the number of (presumably independent) camera traps within a site, or placing two cameras directly opposite one another to record individual identifiers.

Our objective was to evaluate how components of CT study designs influenced detectability of wildlife species by iteratively parsing an empirical dataset over a range of study design parameters. We were specifically interested in investigating how detectability was influenced by (1) the size of a camera array, and (2) season length. We hypothesized that detectability would increase asymptotically with increased number of cameras and season length for species with presumed low detectability. Conversely, we hypothesized that changes in detectability would be negligible for species that are detected frequently within our study sites. We discuss the potential of using multi-camera arrays to improve detection beyond the standard single camera while reducing some of the bias associated with non-random camera placement, particularly in regards to trade-offs between number of cameras and study season length.

## Materials & methods

Our empirical dataset was generated from 40 wildlife cameras (Trophy Cam HD Essential, Bushnell, Kansas) that were deployed as part of an ongoing study of mesopredator activity levels between November 2014 and November 2015 in southeastern Connecticut, USA [15]. Two 1-ha areas were identified in each site and five cameras were deployed at random coordinates [16] in each 1-ha area. Therefore, each of our 4 sites contained 2 5-camera arrays. Cameras were placed at approximately 1 m height, facing away from any large objects or dense vegetation that would severely obstruct the camera image or cause false-trigger events. The distance between cameras within each 1-ha area averaged 65.2∓7.9 m. The average distance between each 5-camera array was 589.3∓137 m. Passive-infrared sensors were set to medium sensitivity, with three images captured per trigger event, and a trigger interval of one minute. We visited cameras monthly to exchange memory cards and batteries. To address independence of camera observations, we defined an observation as a three-photo trigger event containing evidence of wildlife presence, and we mandated that a half-hour had to have passed between observations for them to be considered a unique detection [17–19]. This restriction was applied across all cameras within the site (i.e., 10 total cameras). For example, if a raccoon was observed 10 minutes apart at two different cameras in a site, this was considered a single detection at this site. Observations containing more than one individual of a species were still classified as a binary “presence” for that species in the dataset.

We selected species from our empirical dataset with a range of body sizes and differing degrees of presumed detectability based on known life history traits. White-tailed deer (*Odocoileus virginianus)*, bobcat (*Lynx rufus)*, raccoon (*Procyon lotor)*, and Virginia opossum (*Didelphis virginiana)* detections occurred at least once in every 1-ha area across all sites and within each season during the year-long deployment of cameras. Therefore, we assumed all four species were present at every site throughout the yearlong study and available for detection (i.e., occupancy = 1.0). Failures of CT arrays to capture study species indicated a lack of detection (i.e., false absence) not a lack of occurrence (i.e., a true absence). Consequently, detectability could be calculated directly from zeroes in our detection/non-detection data, rather than inferring detectability as a nuisance variable obscuring true occurrence [7].

Monte Carlo subsampling allowed us to simultaneously evaluate study design components by iteratively parsing data based on the size of an array and season length. As opposed to using simulated detection histories to evaluate study design components, we instead drew specific subsamples of the empirical dataset for each iteration. For each subsample, we randomly selected a start and end date within the calendar year and assumed study years were comparable so any simulated season that extended past Nov 2015 were completed by looping with detection histories beginning in Dec 2014. Although the effect of replicate length (i.e., number of days per survey event) on estimates of detectability was not explicitly evaluated, we recognize that it has implications for occupancy modeling and other applications that require replicated survey events [20]. As opposed to a study design consideration, how researchers choose to separate continuous camera trapping data is an analytical consideration that has been the focus of other studies [21] and should be further evaluated in the context of CT studies. The potential effects of replicate length and number of replicates on detection probabilities were incorporated by randomly selecting a replicate length between 1 and 28 days for each iteration—a range of replicate lengths reported in CT studies [6]. The number of replicates was calculated by dividing season length by replicate length.

Our first objective was to evaluate the effect of array size per site on detection probabilities. We subset the data by randomly selecting an array containing between 1 and 10 cameras per site via Monte Carlo subsampling, and then summed the detections among cameras in the array. Survey detection probabilities were calculated for each subsample using equation 1, p = Σ*P*/(*Nk*), where *p* was the probability of detecting a species during a survey given its presence (henceforth referred to as “survey detection probability”), *P* was either 1 (detection) or 0 (non-detection) based on detection history during a survey replicate, *N* was the number of sites, and *k* was the number of surveys [7]. To evaluate the effect of array size on detection probability, we used Tukey tests for multiple comparisons while accounting for non-normality and heteroscedasticity in the R [22] package *multcomp*. We determined significant differences (p < 0.05) in detection probability between array sizes by generating 5,000 Monte Carlo samples per species.

It is important to distinguish between survey detection probabilities used to investigate the influence of array sizes and the cumulative, season-long probability of detecting a species. For objective two, we were interested in the influence of season length on detection probabilities which was best addressed with cumulative season-long detection probability, for which we used equation 2, p* = 1-(1-*p*)^*k*^, where p* was the probability of detecting a species at least once across all replicate surveys in a season (henceforth referred to as “season detection probability”), *p* was survey detection probability calculated from equation 1, and *k* was the number of surveys. Relationships between season length, array size, and season detection probability were fitted with 10 nonlinear functions for each species with equation 3, p* = *A*(1-*e*^(-*RS*)^), where *A* was the horizontal asymptote of the season detection probability, p*, increasing at a rate of *R* as a function of season length, *S*. Models were fit in R [22] using the base *stats* package and functions *NLS* and *confint*. The University of Connecticut Institutional Animal Care and Use Committee exempted this research from further review (E15-003).

## Results

**Fig 1:**
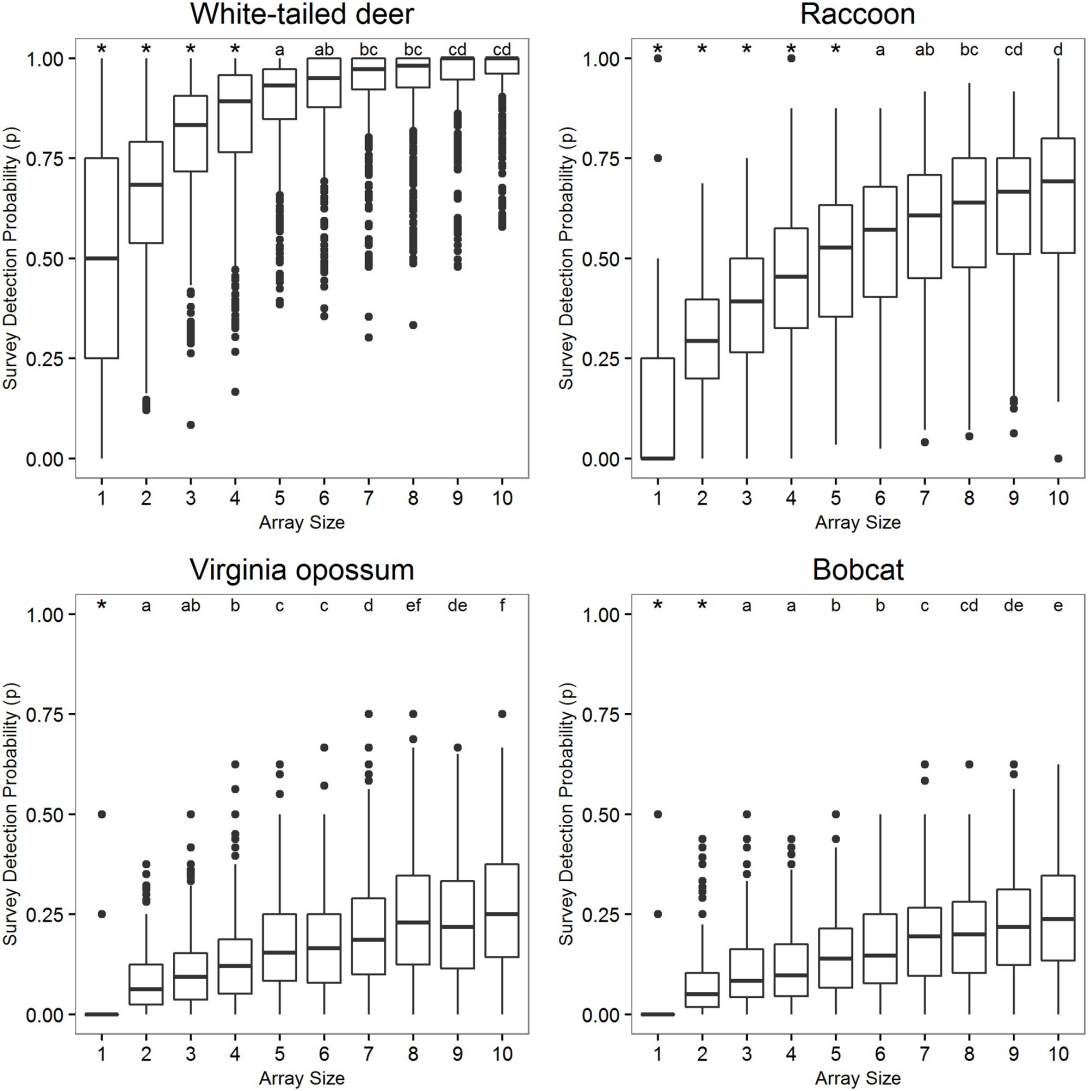
Survey detection probability (p) by array size (number of cameras; 1–10) per site across four species (White-tailed deer, upper left; Raccoon, upper right; Virginia opossum, lower left; Bobcat, lower right), calculated by randomly parsing a yearlong data set by season and replicate length with 5,000 Monte Carlo iterations. Asterisks (*) indicate significant differences and letters indicate non-significance between array sizes based on a multiple comparisons of mean survey detection probability accounting for non-normality and heteroscedasticity (p < 0.05).

Independent observations of all focal species per camera ranged from 11 to 307 with 3,488 total unique detections from November 2014–November 2015. After one year, our four focal species were detected at least once by all 10 cameras within a site. Individual observations typically contained unique detections from only one camera and therefore we did not have to combine observations across cameras to satisfy independence assumptions frequently. Survey detection probability increased with increased array size per site for all four focal species (Fig 1). Regardless of species, increasing the array size from one to two cameras significantly increased detection probabilities (Tukey Multiple Comparisons, p < 0.05; Fig 1). The use of a two camera array increased survey detection an average of 80% (range 40–128%) from the detection probability of a single camera across the four species (Fig 1). For frequently detected species, deer and raccoon, the survey detection probability increased significantly with each camera up to array sizes of five and six, respectively. Alternatively, for infrequently detected species, opossum and bobcat, survey detection probability did not increase significantly with the addition of each camera beyond two, but increasing to array size of eight or nine still had positive effects on detection probability. The array size where adding additional cameras to the array no longer significantly improved survey detectability, was between eight and nine cameras for all four species (Fig 1).

**Fig 2:**
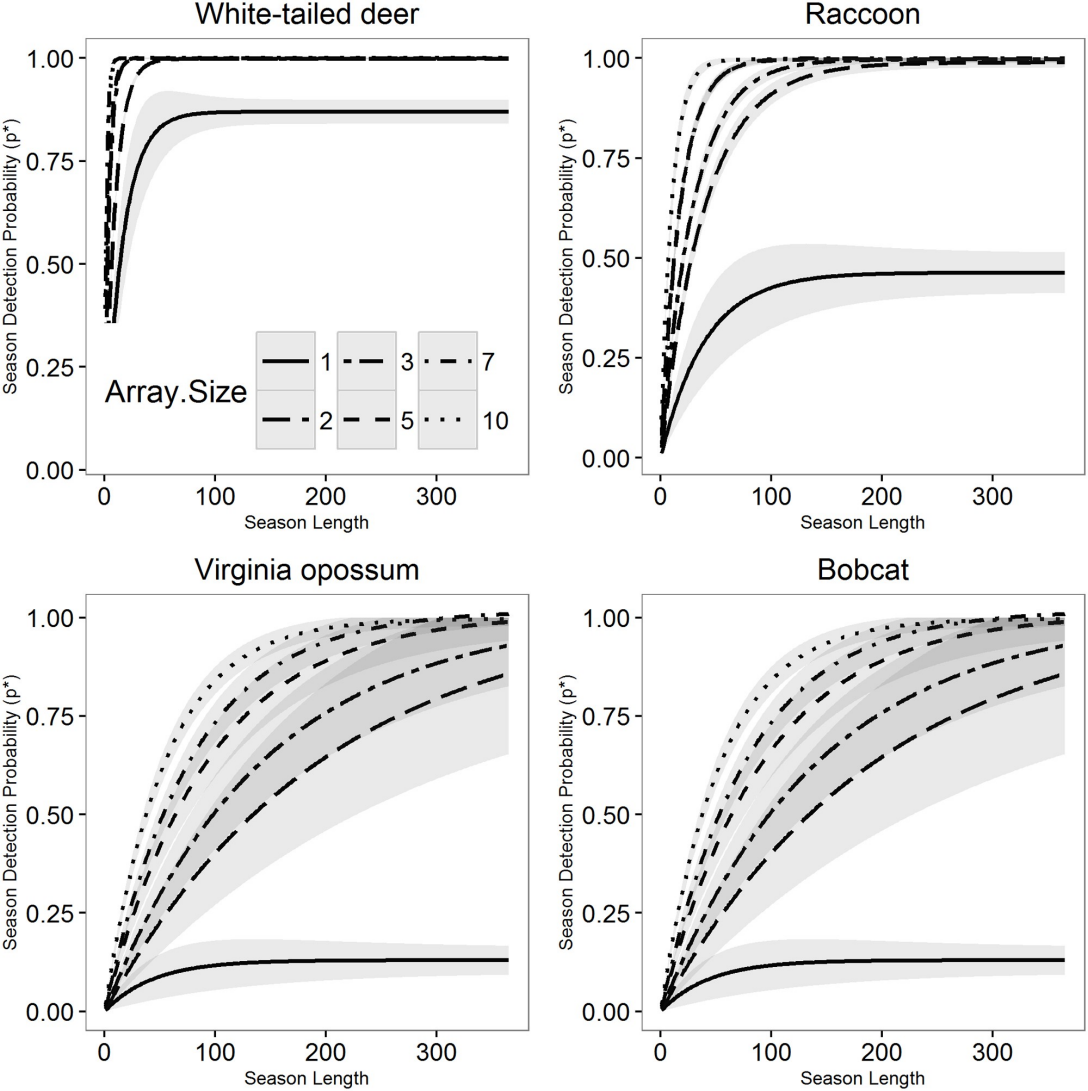
Season detection probability (p*) by season length (0–365 days) and array size (1, 2, 3, 5, 7, 10 cameras; lines) across four species (White-tailed deer, upper left; Raccoon, upper right; Virginia opossum, lower left; Bobcat, lower right), calculated by randomly parsing a yearlong data set with 5,000 Monte Carlo iterations. Lines represent best fit of nonlinear model in R using the *NLS* package and grey indicates 95% confidence intervals estimated from R package *confint*. Only a subset of the total array sizes are displayed to improve visual representation of data.

Increasing season length had a positive effect on season detection probability across all four species and array sizes (Fig 2). However, a minimum array size of two cameras were required to produce detectability curves with 95% confidence intervals overlapping 1.0 (i.e., maximum detectability) for all four species. This point was reached with season lengths of 70 days for deer, 226 days for raccoon, 250 for opossum and 267 days for bobcat. Increasing from a single camera to a two-camera array had the largest effect on season detection probability across the range of season lengths for all four modeled species (Fig 2).

## Discussion

By simulating changes in the study design with empirical CT data, we demonstrated how the use and size of camera arrays and the length of an active survey period can affect detection probabilities. By modifying these two aspects of survey design, survey and season long detection probabilities can be optimized. When only a single camera was used at a site, increasing season length often failed to contribute further increases in season detectability after 100 days. Perfect season detectability was approached when camera arrays were used, although the benefits of multi-camera arrays varied depending on the targeted species of interest. We did not attempt to compare baited vs non-baited camera stations, or random vs non-random camera placement in this study. Previous research suggests that non-random camera placement may negatively influence the robustness of camera data, and that baiting cameras may offer no benefits in increasing detection probability [23,24]. Others have found that any biases associated with non-random camera placement may be irrelevant with enough cameras over a long-term study [25]. Ultimately, we suggest ways to utilize camera arrays that reach high detection probabilities and eliminate the use of bait and non-random camera placement. Our findings can guide future CT studies by providing suggestions for resource allocation based on specific research goals and objectives.

Desired survey or season probabilities of detection will vary by target species and study objectives, but researchers should establish robust study designs based around achieving *a priori* defined detection probabilities. Minimum thresholds of detectability may be particularly relevant for occupancy studies of species that are detected infrequently by remote cameras. Perfect detection is unreasonable to expect in natural systems and is an inefficient study design goal. Statically robust estimates of metrics such as occupancy can be made with survey detection probabilities (*p*) < 1.0 [26]. However, single species data with low survey detection probabilities (*p* < 0.15) and/or limited detection histories (i.e., few survey replicates) may lead to highly uncertain occupancy parameters that provide poor foundations on which to make inferences [7, 12, 27, 28]. Probabilities of survey detection > 0.40 are generally more than adequate for occupancy or abundance estimation [12, 29]. For opossum and bobcat, two species with lower numbers of detections, increasing array sizes to ten still did not reach a survey detection probability of 0.40. The constraints on inference that arise from low detection probabilities extend to other methods of collecting detection/non-detection data [21, 26, 27] rather than being unique to CT studies.

Camera trap studies often attempt to boost low survey detection probabilities, and therefore season-long probabilities, by using non-random camera placement or baited camera sites, which can lead to higher detection probabilities but biased data. Suggested targets for season-long detection probabilities of focal species are less well established, but have meaningful implications on the effort requirements (e.g., labor, budget) of a study. While season-long detection reached probabilities of > 0.8 in some of our modeled scenarios, particularly by using arrays with large numbers of cameras, we consider this degree of detection excessive and unnecessary for a real-world CT study design to achieve. Inserting the minimum recommended survey detection probability of 0.15 [30] and suggestion of three-replicate study designs for occupancy modeling [10, 12] into equation 2, these criteria would result in a minimum season-long detectability of 0.39. However, acceptable levels of survey detection probability should be explicitly addressed on a per-study and per-species basis in relation to the research question of interest.

Single cameras used in CT studies are limited in their ability to achieve high season-long detectability of small-bodied or cryptic wildlife species. Improving season-long detectability for these species is particularly important if study objectives involve evaluating species richness or biodiversity [28], i.e., detecting as many species within a given area as possible, because lack of *a priori* consideration for detection probabilities would negatively bias these estimates. For two of our modeled species, Virginia opossum and bobcat, data from a single camera produced maximum season-long detectabilities of 0.13 and 0.14, respectively, even after 180 days. The addition of a second, randomly-placed camera to create an array increased season detectability over a 180-day survey to 0.86 and 0.54, respectively. In addition to improved detection probabilities, the inclusion of a second camera to the array likely reduced the risk of lost data or failure to detect a species due to camera malfunction [1, 31]. While an intensive design with large camera arrays may be unnecessary for some research objectives or scenarios, they may provide utility for those researchers attempting to survey difficult to detect wildlife.

The use of camera arrays as opposed to single cameras per site can vastly improve data quality without the need to resort to biased sampling designs. For example, bobcat are a frequent target of CT studies, have naturally low densities throughout their range (e.g., 0.25–0.42 bobcat/km2 reported by [32]), and are almost universally reported as having low detection probabilities (e.g., survey detectability ≤0.027 reported by [33]), when detected at all [18, 34, 35]. In our study, relatively high season long detection probabilities (>0.50) for bobcat and Virginia opossum were always reached when arrays of ≥2 cameras were used with season lengths >200 days. Camera arrays consistently resulted in higher detection probabilities but there were specific scenarios when single cameras remained appropriate. Single cameras have been commonly used in published CT studies and are likely chosen to maximize spatial coverage of a study [4]. In parsing our data to represent a single-camera study design, high probabilities of detection were achieved in very short study seasons for a large-bodied and relatively frequently detected wildlife species in our region, the white-tailed deer (e.g., 30–80 deer/km2 reported by [36]). The likelihood of detecting white-tailed deer, given the known presence of the species at a site, exceeded 75% in only 30 days using a single-camera array (Fig 2). Knowledge of deer presence may be crucial for informing game management practices in both urban and rural settings and CT technology has been proposed as a way to reduce bias and effort in surveying local deer populations [37, 38]. In these situations, single cameras are seemingly adequate in achieving high season long detection probabilities of a target species.

Long-term deployment of cameras using single cameras are perhaps the most frequent way that researchers attempt to increase detectability, particularly if they are limited by the total number of cameras available. This increases study length and results in reported seasons that can last to more than a year [6]. CT methods typically quantify effort in camera-days and have therefore implicitly assumed that camera-days are equal; which would mean that one camera deployed for a long period of time is equal to multiple cameras deployed for a shorter study period. Our simulated scenarios indicated that increasing season length did not always result in meaningful increases in detectability, and that where increases occurred, they were not equal to those achieved by increasing array size (Fig 2). While season-long detectability curves for our simulated arrays sometimes reached asymptotes quickly (usually around a 100-day season), for single cameras, these asymptotes were <0.15 for our two focal species with relatively low probabilities of detection. Increasing season length to a year for these difficult to detect species failed to provide increased detectability benefits when using a single camera. Committing resources to longer field seasons for these species would have resulted in relatively little return on investment. Shorter seasons did not result in the sacrifice of meaningful detection data and may be preferable when aiming to collect CT data as efficiently as possible. Rather than forming study objectives around the detection of as many species as possible, researchers may benefit from either designing studies specific to the targeted species or, alternatively, considering those species that are known to be difficult to detect when designing CT studies.

Aside from issues of efficiency, increasing season length and treating excessively long seasons as a single survey has meaningful implications when addressing assumptions of closure, i.e., no changes in occupancy between surveys [39]. Employing large season lengths in an attempt to achieve sufficient detection probabilities may mask patterns of seasonal occupancy in species with large home ranges that are capable of traveling large distances over the course of a year. Even if a species does occur within a site continuously over long time periods, detectability may vary substantially between seasons based on changes to vegetation structure and changes in animal behaviors [27]. Long seasons may be appropriate if the intent is to study changes in patterns from one season to the next, but less appropriate for judging presence or absence of a species within a site because of violations of model assumptions. In our study, we assessed detection probabilities over a range of season lengths, ranging from 2 to 365 days. While it is possible that assumptions of closure are violated within a yearlong study, we chose to include the maximum possible range of season lengths based on our data to reflect the broad ranges reported in the CT literature [6]. Regardless, closure assumptions are closely tied to the specific questions being addressed in a study and the ecology of the species involved [40]. A study design that was highly efficient for detecting deer, requiring few cameras and minimal time investment, would have resulted in low detection of bobcat (Fig 2)

Researchers must balance the desire to maximize overall detection probability and spatial coverage given a limited number of cameras and days available for their study. The change from a single camera to even a two-camera array will likely increase detectability during the season but would reduce the number of sites being sampled by half. However, the increase in both survey and season detection probability over short season lengths could allow researchers to retrieve and relocate cameras, thus achieving greater spatial coverage of a landscape without sacrificing data quality. As a hypothetical scenario, if a researcher has 40 cameras and a 90-day field season, they would choose an array size which allows them to reach some desired minimum season detection probability, say p* ≥ 0.4. A traditional camera trap design with one camera per site would maximize the study’s spatial coverage but would also result in low detection probabilities that would limit the ecological inference that could be made for Virginia opossum and bobcat because they never reached p* > 0.2. A three-camera array would increase season detection probabilities by 412% and 343% for Virginia opossum and bobcat, respectively, reaching the season detection probability minimum but also reduce the number of sites by a third. However, given a minimum season detection probability of 0.4, three-camera array confidence intervals surpassed this target within 55 days for both Virginia opossum and bobcat, which could enable the researcher to relocate the arrays and increase their spatial coverage to 32 sites if the study season could be extended to 110 days. These results suggest that projects with a limited number of cameras may want to consider whether their research question can be answered with a study design that uses camera arrays over short time periods and move arrays to cover the desired spatial extent.

Efficiency and rigor of data collection is a common goal for researchers and managers. Randomized CT arrays containing two or more cameras can greatly improve detection probabilities (*p* ≥ 0.15) within relatively short season lengths, which improves upon single-camera study designs maintained over extensive timeframes. In addition to achieving greater overall efficiency, camera arrays have the potential to dramatically improve the detectability of cryptic and difficult-to-detect species over a shorter study period without resorting to biased sampling designs. CT technology already shows tremendous utility in collecting wildlife data in a manner that is minimally-invasive and requires reduced human labor. Combining this methodology with a clearer justification of study design can result in rigorous data collection and a broadly applicable tool for research and management.
